# Vaccine coverage and effectiveness against laboratory-confirmed symptomatic and severe Covid-19 in indigenous people in Brazil: a cohort study

**DOI:** 10.1186/s12889-023-16196-4

**Published:** 2023-06-29

**Authors:** Julia M. Pescarini, Andrey M. Cardoso, Ricardo Ventura Santos, Priscila F. Scaff, Enny S. Paixao, Otavio T. Ranzani, Thiago Cerqueira-Silva, Viviane S. Boaventura, Juracy Bertoldo-Junior, Vinicius A. de Oliveira, Guilherme L. Werneck, Mauricio L. Barreto, Manoel Barral-Netto

**Affiliations:** 1 Center of Data and Knowledge Integration for Health (Cidacs), Fiocruz, Salvador, BA Brazil; 2 London School of Hygiene and Tropical Medicine, London, UK; 3 Escola Nacional de Saúde Pública (ENSP), Fundação Oswaldo Cruz, Rio de Janeiro, RJ Brazil; 4 Museu Nacional, Universidade Federal do Rio de Janeiro, Rio de Janeiro, Brazil; 5 Barcelona Institute for Global Health, Universitat Pompeu Fabra (UPF), CIBER Epidemiología y Salud Pública (CIBERESP), ISGlobal, Barcelona, Spain; 6 Pulmonary Division, Heart Institute, Faculty of Medicine, Hospital das Clínicas da Faculdade de Medicina da Universidade de São Paulo, São Paulo, Brazil; 7 Universidade Federal da Bahia (UFBA), Salvador, BA Brazil; 8 LIB and LEITV Laboratories, Instituto Gonçalo Moniz, Fiocruz, Salvador, BA Brazil; 9 Universidade do Estado do Rio de Janeiro, Rio de Janeiro, RJ Brazil; 10 Universidade Federal do Rio de Janeiro (UFRJ), Rio de Janeiro, RJ Brazil

**Keywords:** COVID-19, Vaccine effectiveness, Vaccine coverage, Cohort study, Indigenous peoples, Brazil

## Abstract

**Background:**

Indigenous people have historically suffered devastating impacts from epidemics and continue to have lower access to healthcare and be especially vulnerable to respiratory infections. We estimated the coverage and effectiveness of Covid-19 vaccines against laboratory-confirmed Covid-19 cases among indigenous people in Brazil.

**Methods:**

We linked nationwide Covid-19 vaccination data with flu-like surveillance records and studied a cohort of vaccinated indigenous people aged ≥ 5 years between 18th January 2021 and 1st March 2022. We considered individuals unexposed from the date they received the first dose of vaccine until the 13th day of vaccination, partially vaccinated from the 14th day after the first dose until the 13th day after receiving the second dose, and fully vaccinated onwards. We estimated the Covid-19 vaccination coverage and used Poisson regression to calculate the relative risks (RR) and vaccine effectiveness (VE) of CoronaVac, ChAdOx1, and BNT162b2 against Covid-19 laboratory-confirmed cases incidence, mortality, hospitalisation, and hospital-progression to Intensive Care Unit (ICU) or death. VE was estimated as (1-RR)*100, comparing unexposed to partially or fully vaccinated.

**Results:**

By 1st March 2022, 48.7% (35.0-62.3) of eligible indigenous people vs. 74.8% (57.9–91.8) overall Brazilians had been fully vaccinated for Covid-19. Among fully vaccinated indigenous people, we found a lower risk of symptomatic cases (RR: 0.47, 95%CI: 0.40–0.56) and mortality (RR: 0.47, 95%CI: 0.14–1.56) after the 14th day of the second dose. VE for the three Covid-19 vaccines combined was 53% (95%CI:44–60%) for symptomatic cases, 53% (95%CI:-56-86%) for mortality and 41% (95%CI:-35-75%) for hospitalisation. In our sample, we found that vaccination did not reduce Covid-19 related hospitalisation. However, among hospitalised patients, we found a lower risk of progression to ICU (RR: 0.14, 95%CI: 0.02–0.81; VE: 87%, 95%CI:27–98%) and Covid-19 death (RR: 0.04, 95%CI:0.01–0.10; VE: 96%, 95%CI: 90–99%) after the 14th day of the second dose.

**Conclusions:**

Lower coverage but similar Covid-19 VE among indigenous people than overall Brazilians suggest the need to expand access, timely vaccination, and urgently offer booster doses to achieve a great level of protection among this group.

**Supplementary Information:**

The online version contains supplementary material available at 10.1186/s12889-023-16196-4.

## Background

Indigenous peoples have historically suffered the devastating impacts of epidemics of infectious diseases that have resulted in drastic reductions in their population over the centuries [1, 2]. The high risk of infectious diseases, including acute respiratory infections [3–6], is largely attributed to poverty, precarious sanitation, and limited access to health care. In Brazil [7, 8], this is further exacerbated by the long history of exposure to discrimination, violence, environmental degradation, and territorial restriction [2, 3, 9], which perpetuate respiratory infection as a major health issue for indigenous populations [3–6].

The Covid-19 pandemic has disproportionally impacted socially disadvantaged population groups in Brazil, including indigenous peoples [10–13]. In the first trimester of the pandemic, there was a rapid increase in the risk of sustained transmission of Covid-19 in areas with an indigenous presence [11]. Covid-19 was initially concentrated in large urban centres of Brazil and their surroundings, such as state capitals and metropolitan regions. However, there was a rapid internalization of the disease especially in the North region, facilitated by intense population circulation through the large rivers, and later reaching the countryside of the Central-West region, placing the indigenous territories a high risk for the introduction of the disease [11]. Two national household surveys of seroprevalence of antibodies against SARS-CoV-2 in 133 cities showed an 87% higher adjusted prevalence among indigenous subjects than whites [10]. In addition, in the first year of the pandemic, mortality among indigenous people was 16.7% higher than that observed in the general Brazilian population [12]. Furthermore, age-specific mortality rates [14] and hospital case fatality rates in all age groups were also higher in indigenous subjects compared to other colour/race categories registered in Brazilian health information systems [13, 14]. The social vulnerability and the severe impact of the pandemic on the indigenous peoples resulted in the inclusion of the population covered by the Brazilian Indigenous Health Care Subsystem (IHS) as one of the priority groups for vaccination against Covid-19 [15, 16].

Although two of the main vaccines available in Brazil, ChAdOx-1 (previously Vaxzevria/Fiocruz or Oxford-AstraZeneca) and CoronaVac/Butantan, proved to be effective (i.e., > 50%) in protecting against SARS-CoV-2 symptomatic infection and severity, lower vaccine effectiveness (VE) was seeing in some population strata, such as elderls [17]. VE is also likely to change according to intersecting factors, such as the type of vaccine and vaccination schedule, comorbidities, risk of exposure, time after vaccination, and circulation of specific variants [18]. To our knowledge, no investigation has been carried out about VE against Covid-19 in the Indigenous populations in Brazil. In this study, we estimated the coverage of Covid-19 vaccines and evaluated VE against infection, hospitalisation, admission to ICU, and death related to SARS-CoV-2 in the indigenous population in Brazil. We provide crucial evidence to be considered in health policies aimed at mitigating ethnic-racial gaps in health in the country.

## Methods

### Study design and setting

We followed a cohort of Covid-19 vaccinated indigenous individuals living in municipalities that overlap with the 34 Special Indigenous Health Districts *(Distritos Sanitários Especiais Indígenas*, DSEIs), which provide primary health care to indigenous living in villages [19, 20] DSEIs are a service-oriented organisation model, based on the geographical occupation of indigenous communities that promotes targeted ethnocultural, geographic and population-specific healthcare activities [19, 20].

In Brazil, Covid-19 vaccination was started on 18th January 2021 by the National Immunization Program (PNI) of the Brazilian Ministry of Health. It initially relied on the CoronaVac-Sinovac/Butantan, the first vaccine in the country, and the ChAdOx-1 vaccine. Later, Brazil implemented vaccination with BNT162b2 (Pfizer/BioNTech) and Ad26.COV2.S (Janssen). Covid-19 vaccination followed a pre-specified calendar, including in the campaign’s first phase, elderly, healthcare professionals, and indigenous individuals attended by the DSEIs of the IHS. This population mainly comprises subjects living in Indigenous Lands in rural areas. It also includes a smaller proportion of indigenous subjects residing in Indigenous Lands in urban areas or outside indigenous lands, both in urban and rural areas. In September 2021, the government initiated the vaccination of adolescents aged 12 to 18 years, and in January 2022, of children from 5 to 11 years.

### Data sources

We used data from (i) individuals vaccinated for Covid-19 from the Information System of Brazilian National Vaccination Programme (SI-PNI) and SARS-CoV-2 using (ii) laboratory-confirmed cases of symptomatic Influenza-like Illness (ILI) notified in the Brazilian Influenza-like surveillance information system (*e-SUS-Notifica*) and (iii) laboratory-confirmed cases of Severe Acute Respiratory Infection (SARI) notified in the Flu Epidemiologic Surveillance System (SIVEP-Gripe), from Brazil’s Unified Health System. Datasets were extracted on 1st March 2021 and made available by the Brazilian Ministry of Health. The information technology bureau of the Brazilian Ministry of Health provided pseudo-anonymised data with a common unique identifier that was used to link individual-level records from the three databases (more details about linkage procedures are available at https://vigivac.fiocruz.br/). Although similar individual datasets are publicly available, the one containing the common unique identifier was provided to our team under authorisation from the Brazilian Ministry of Health and after ethical approval from the Brazilian National Commission on Research Ethics (CONEP). The linkage was performed in a secure environment where it underwent complete disidentification before it was analysed by the researchers.

From the SI-PNI dataset, we extracted information on individuals’ age, sex, municipality of residence, the date of the first and second doses, and the type of vaccine received. From e-SUS-Notifica (ILI cases) and SIVEP-Gripe (SARI cases) databases, we extracted information on age, sex, date of first symptoms, notification date, date of admission to hospital and Intensive Care Unit (ICU), and outcomes of interest: hospitalisation, ICU admission or death. In addition, we used the municipality-level material deprivation index (Brazilian Deprivation Index - *Índice Brasileiro de Privação*, IBP) that combines information on income, education and living conditions from the 2010 Brazilian Census [21] as a *proxy* of the municipal socioeconomic context of where the indigenous community is located.

### Study population

We identified indigenous individuals five years of age and older vaccinated between 18th January 2021 and 1st March 2022. For the vaccine effectiveness (VE) estimation, we excluded (i) individuals who received vaccines other than CoronaVac, ChAdOx-1, or BNT162b2, (ii) individuals who received two heterologous doses of any vaccine, (iii) individuals with ILI or SARI within 90 days before starting vaccination, and (iv) individuals with two doses within < 14 days.

### Exposure and outcomes

We followed individuals from the date of receipt of the first dose until the date they presented each of the outcomes (i.e., symptomatic Covid-19 laboratory confirmed by PCR or antigen test, hospitalisation, admission to ICU or death for Covid-19), or until 1st March of 2022, date of the end of the study, whichever came first. For individuals who received the third dose, we also restricted their follow-up to the date they receipt that dose. As this study comprised a cohort of vaccinated individuals, we considered individuals as (i) unexposed from the date they received the first dose of vaccine until the 13th day of vaccination; (ii) partially vaccinated from the 14th day after the first dose until the 13th day after receiving the second dose; or (iii) fully vaccinated from the 14th day after the second dose onwards.

ILI cases were defined by fever and cough or sore throat. SARI cases were characterised by fever, cough, shortness or difficulty breathing, and hospitalisation or, for those not hospitalised who were notified of death for Covid-19. Laboratory-confirmed ILI or SARI included those with a positive PCR or antigen test for COVID following the first ten days of the start of the symptoms. We considered the primary outcome (i) a case of ILI or SARI due to laboratory-confirmed Covid-19, and secondary outcomes (ii) hospitalisation for Covid-19, (iii) death for Covid-19, and (iv) admission to the Intensive Care Unit (ICU) and (v) death for Covid-19 in hospitalised patients.

### Statistical analysis

**Fig. 1:**
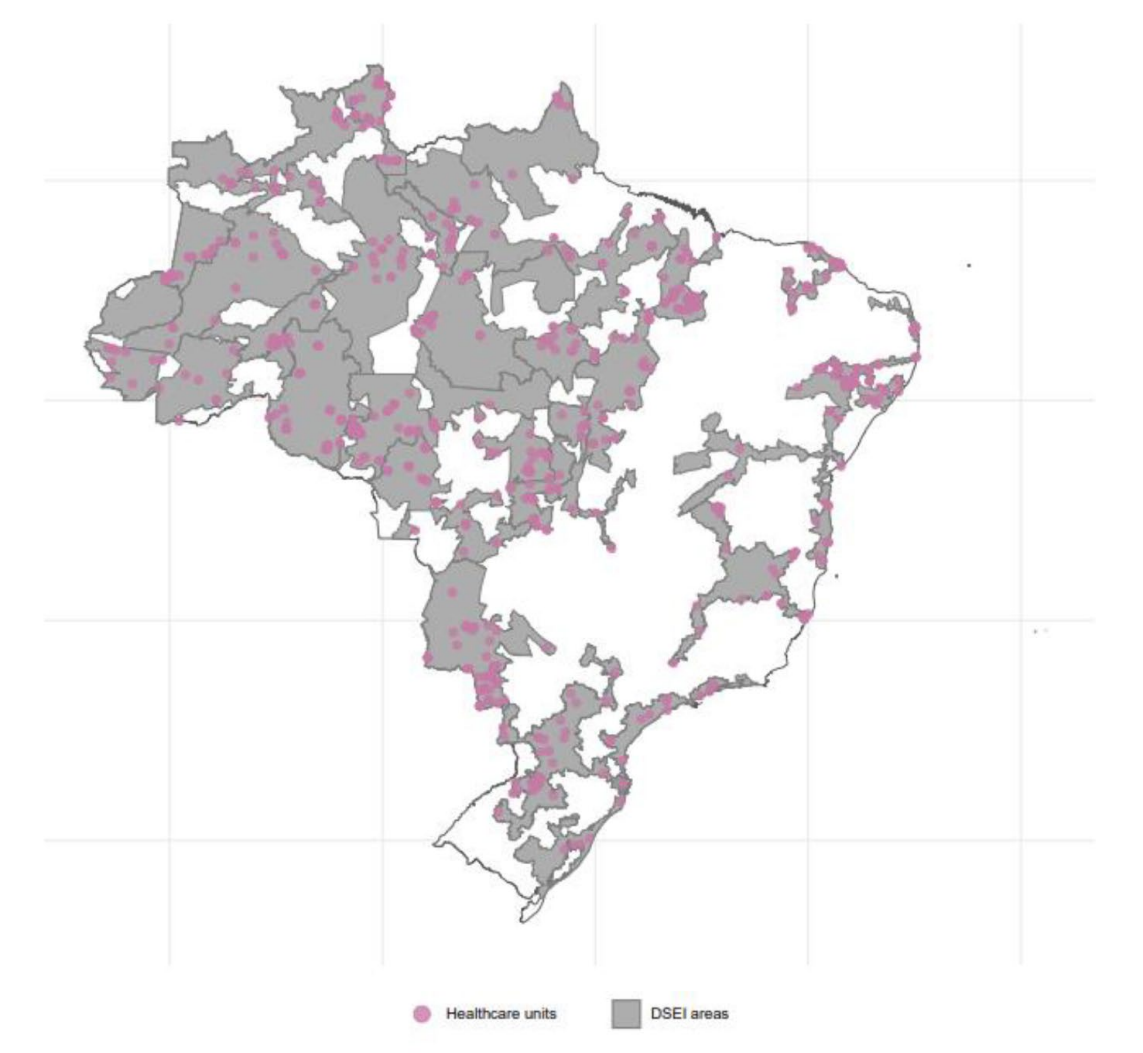
Distribution of healthcare units that overlay with Special Indigenous Health Districts *(Distritos Sanitários Especiais Indígenas*, DSEIs) in Brazil. *Pink points show data on 954 out of 1022 (93.3%) healthcare units with geocoded data*[32]; *In grey, we show DSEIs areas (data available at Fundação Nacional dos Povos Indígena*[33]

We estimated vaccination coverage for indigenous people living in municipalities overlapping with the DSEI territories (See Fig. 1) by age, sex, region of residence, and IBP. To do this, we first had to estimate the indigenous demographic distribution for the year 2020. In Brazil, the official socio-demographic data result from the decennial Demographic Censuses and population counts that have been carried out since 1940 by the Brazilian Institute of Geography and Statistics (IBGE)(Brazilian Institute of Geography and Statistics [IBGE], 2022). Since the most recent official estimate of the indigenous population in Brazil is from the 2010 Demographic Census, we developed a strategy to estimate the indigenous population in 2020 for further calculation of Covid-19 vaccine coverage.

First, we estimated the number of indigenous people in 2020 living in municipalities that overlap with the DSEIs territories (480/5,570) by age and sex strata. For reasons related to statistical confidentiality, the number of subjects enumerated in the National Census, which also applies to the indigenous population, is not provided in data outputs when they are from 1 to 6 individuals. To circumvent this possible inaccuracy in the size population, we obtained the number of indigenous people aged five or more in 2010 by calculating the difference between the total population in this age range minus the total non-indigenous population in the same age range for each municipality. Then, for each municipality, we estimated the size of the indigenous population in 2020 by multiplying the percentage of the indigenous population in 2010 in each age and sex stratum by the overall 2020 population estimates provided by IBGE. Finally, we calculated vaccine coverage for partial and full vaccination as the number of individuals who received either (i) one dose of CoronaVac, ChAdOx-1, or BNT162b2 (partial), or (ii) one dose of Ad26.COV2.S or two doses of CoronaVac, ChAdOx-1 or BNT162b2; divided by the number of indigenous individuals estimated for 2020 within each stratum of age and sex, later grouped by region of residence and municipal deprivation level. We used as a numerator the total number of doses before applying exclusion criteria, i.e., considering individuals receiving Janssen or a mixed calendar, symptoms before the vaccine, and inconsistencies between doses. The difference in the proportion of vaccinated indigenous and the total population was calculated using Z-test and their respective p-value considering a 5% error.

To evaluate VE against each outcome, we performed Poisson regression using the follow-up time as the offset to calculate the relative risks (RR) and 95% Confidence Intervals (95%CI) for individuals partially or fully vaccinated, relative to non-exposed individuals. We analysed any of the three combined vaccination schemes (CoronaVac, ChAdOx-1, or BNT162b2) and exclusively for CoronaVac. Vaccine effectiveness for Ad26.COV2.S was not performed as the definition of partial or fully immunised differ from the remaining vaccines. Our analysis considered that each individual contributed first as unexposed and later as partially or fully vaccinated. We included cluster robust standard errors to account for individuals contributing to multiple rows. The analysis was adjusted for age, sex, region of residence, IBP, and the month of the first dose. Age was used as a continuous variable, given the sample size. Models evaluating VE for Covid-19 mortality, hospitalisation, and hospital-based admission to ICU or death were only adjusted for age or age and sex to avoid over-adjustment. VE was estimated as (1-RR)*100.

In addition, to estimate if VE changed by year after the introduction of Omicron variant in Brazil, we estimated VE restricting it to individuals who received the first dose up to 31st December 2020 and followed the individuals up to the same date. This analysis considered that from January 2021, more than 50% of the cases were due to this variant [22].

All analyses were performed in STATA 17.0 (Serial number 401,709,302,491), and visualisation was performed with R Studio (version 4.2.2; packages geobr, sf, ggplot2, and ggmap).

## Results

**Fig. 2:**
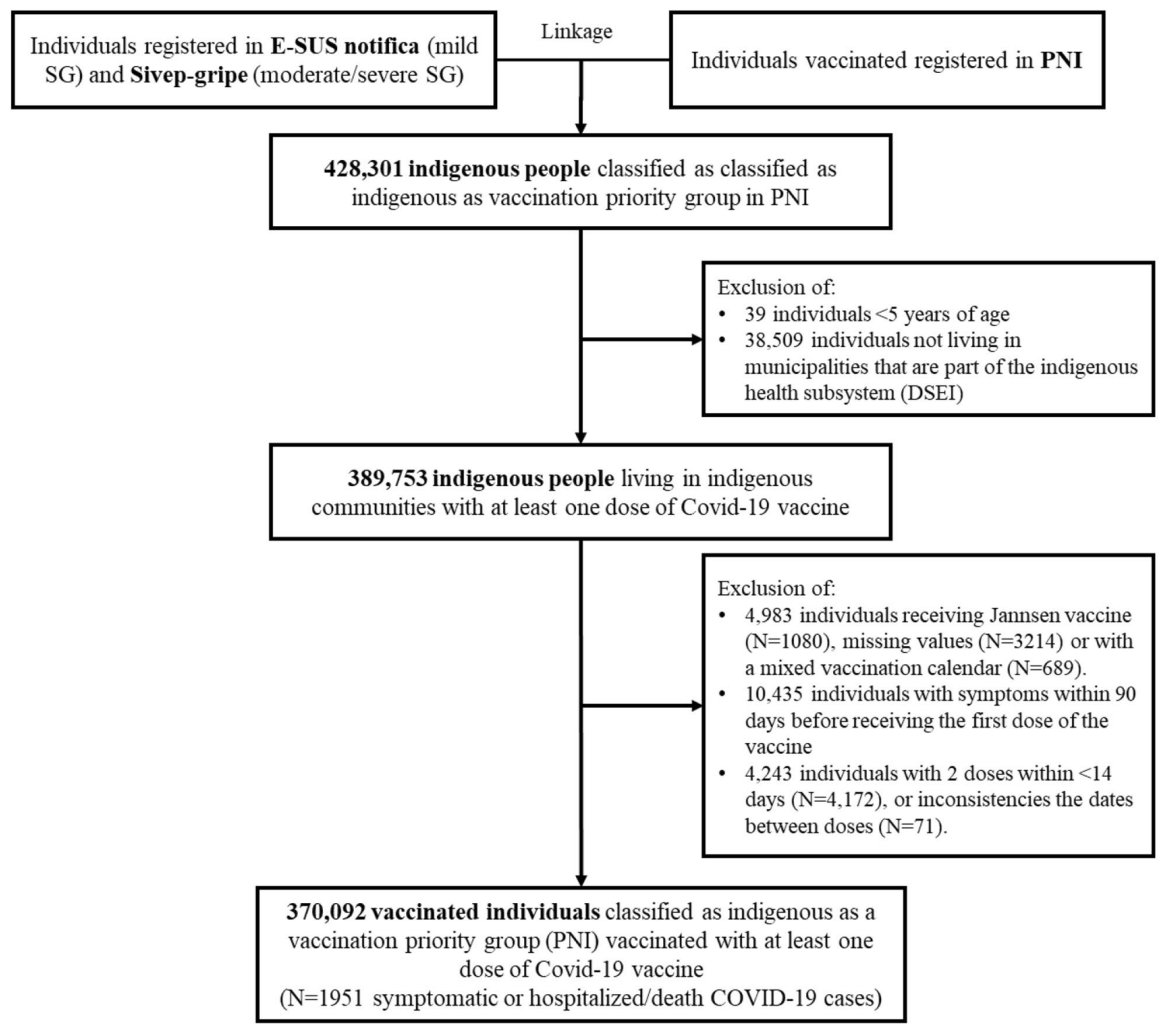
Flowchart of data selection of the cohort of indigenous people living on indigenous land and vaccinated with Covid-19 vaccines

Using the data of 389,753 eligible indigenous people (Fig. 2), the overall vaccine coverages were 65.0% and 48.7% for partial or full vaccination (Table 1). Coverage of over 90% was achieved only for partial vaccination in adults in all age strata over 20 years old. The highest percentage of full vaccination occurred among those aged 50 to 59 years (77.2%). Among people aged 10–19 years, partial and full vaccination coverages were 40.7% and 21.3%, respectively, and below 3% in children 5–9 years (Table 1).

**Table 1:** Vaccine coverage among people aged five or more overall and among the indigenous living in municipalities overlapping DSEIs territories (target of priority for vaccination) according to sex, age, region, and deprivation index quintiles in Brazil up to 1st March 2022

Characteristic	Coverage partial vaccination^a^ (% and 95% CI)		Coverage full vaccination^b^ (% and 95% CI)	
	Overall (Brazil)	Indigenous living in municipalities overlapping with the DSEIs	Overall (Brazil)	Indigenous living in municipalities overlapping with the DSEIs
**Overall**	87.6 (69.3–106.0)	65.0 (49.2–80.8)	74.8 (57.9–91.8)	48.7 (35.0-62.3)
**Sex**				
Woman	89.3 (70.8-107.9)	65.6 (49.7–81.5)	77.5 (60.3–94.8)	49.6 (35.8–63.4)
Men	85.8 (67.6-103.9)	64.4 (48.7–80.2)	72.0 (55.4–88.6)	47.8 (34.2–61.3)
**Age (years)**				
5–9	40.7 (28.2–53.2)	2.6 (-0.6-5.8)	2.0 (-0.8-4.8)	0.0 (-0.3-0.3)
10–19	81.5 (63.8–99.2)	40.7 (28.2–53.2)	51.6 (37.5–65.7)	21.3 (12.2–30.3)
20–49	89.6 (71.1-108.2)	96.9 (77.6-116.2)	81.8 (64.1–99.5)	77.2 (60.0-94.4)
50–59	97.1 (77.8-116.4)	101.1 (81.4-120.8)	92.3 (73.5-111.2)	83.1 (65.3–101.0)
#x003E; 60	102.5 (82.6-122.3)	90.8(72.1-109.4)	97.3 (78.0-116.7)	74.7 (57.7–91.6)
**Region**				
North	84.6 (66.6-102.6)	56.9 (42.1–71.6)	73.7 (56.9–90.5)	40.3 (27.8–52.7)
Northeast	85.5 (67.4-103.7)	80.4 (62.8–97.9)	70.3 (53.8–86.7)	66.4 (50.5–82.4)
Southeast	76.3 (59.2–93.5)	47.9 (34.3–61.4)	60.8 (45.5–76.1)	41.0 (28.5–53.6)
South	89.8 (71.2-108.4)	68.9 (52.6–85.1)	77.9 (60.6–95.2)	43.0 (30.1–55.8)
Central-West	89.9 (71.3-108.5)	73.0 (56.3–89.8)	80.8 (63.2–98.4)	56.3 (41.6–71.0)
**IBP quintiles**				
1 (less deprived)	90.3 (71.7-108.9)	16.7 (8.7–24.7)	80.2 (62.6–97.8)	9.1 (3.2–15.1)
2	89.6 (71.0-108.1)	30.7 (19.8–41.5)	77.9 (60.6–95.2)	23.3 (13.8–32.8)
3	87.5 (69.2-105.8)	95.2 (76.1-114.3)	75.5 (58.5–92.6)	71.6 (55.0-88.2)
4	87.0 (68.7-105.3)	81.8 (64.1–99.6)	73.8 (56.9–90.6)	59.7 (44.6–74.8)
5 (more deprived)	82.0 (64.3–99.8)	62.2 (46.7–77.6)	66.3 (50.3–82.2)	46.9 (33.5–60.3)

^a^Partial vaccination - one dose of ChAdOx-1, CoronaVac or BNT162b2.

^b^Full vaccination - two doses of ChAdOx-1, CoronaVac, or BNT162b2; or one dose of Jannsen

VE was estimated among 370,092 indigenous subjects who remained in the study after applying the exclusion criteria (Fig. 1). The most frequent vaccine received was CoronaVac (322,102 doses; 87.0%), followed by BNT162b2 (43,795 doses; 11.8%) and ChAdOx-1 (4,266 doses; 1.2%). 75% (262,081/370,092) received a second dose of the vaccine (Table 2). Older indigenous individuals and those living in the Southeast or Northeast or more deprived municipalities were more likely to have received the second dose.

**Table 2:** Data for indigenous people who received at least one dose of Covid-19 vaccine according to whether they received one or two doses of ChAdOx-1, CoronaVac, or BNT162b2.

Covariates	1 dose n = 108,011	2 doses n = 262,081	Total n = 370,092	p-value^1^
	N (row%)	N (row%)	N	
**Sex**				< 0.001
Woman	52,279 (28.6)	130,742 (71.4)	18,321	.
Men	55,732 (29.8)	131,339 (70.2)	18,771	.
**Age**				< 0.001
5 to 9 years	2,612 (99.3)	18 (0.7)	2,630	.
10 to 19 years	45,961 (69.5)	20,173 (30.5)	66,134	.
20 to 49 years	47,237 (20.5)	183,417 (79.5)	230,654	.
50 to 59 years	5,701 (17.6)	26,642 (82.4)	32,343	.
60 or more	6,500 (17.0)	31,831 (83.0)	38,331	.
**Region**				< 0.001
North	51,416 (32.0)	109,141 (68.0)	160,557	.
Northeast	22,348 (22.7)	76,022 (77.3)	98,370	.
Southeast	3,200 (21.3)	11,835 (78.7)	15,035	.
South	13,347 (41.7)	18,696 (58.3)	32,043	.
Central-West	17,700 (27.6)	46,387 (72.4)	64,087	.
**Deprivation index (IBP quintiles)**				< 0.001
1 (less deprived)	685 (52.5)	619 (47.5)	1,304	.
2	1,712 (32.5)	3,561 (67.5)	5,273	.
3	4,943 (30.4)	11,306 (69.6)	16,249	.
4	25,968 (32.2)	54,786 (67.8)	80,754	.
5 (more deprived)	74,703 (28.0)	191,809 (72.0)	266,512	.

^1^P-value for the difference in proportion using Z-test

There were 1951 Covid-19 cases during the study period. One hundred five of them were hospitalised (5.4%), of which 37 were further admitted to ICU (35.2%), and 35 died (1.8% of all symptomatic and 33.3% of hospitalised cases). Age-adjusted effectiveness of the 1st dose of jointly CoronaVac, ChAdOx-1, or BNT162b2 schemes (partial vaccination) against Covid-19 incident cases was 55% (95%CI:46–63%), and 47% (95%CI: 37–55%) after the second dose (full vaccination) (Table 3). After adjusting for sex, time of vaccination, region, and municipal deprivation index (IBP), VE against laboratory-confirmed incident cases was 51% (95%CI:41–60) for partial vaccination and 53% (95%CI:44–60) for full vaccination. After the second dose, age and sex-adjusted VE was 53% (95%CI:-56-86%) against mortality and 41% (95%CI:-35-75) against hospitalisation. Among hospitalised patients, the age-adjusted VE was 87% (95%CI:14–98) for progression to ICU and 96% (95%CI:90–99) for progression to death. We obtained similar point estimates when restricting the analysis to indigenous people vaccinated with CoronaVac (Table 3), and by restricting the analysis to 361,900 (97.8%), indigenous people vaccinated with the first dose in 2021 and followed up to the end of that year (Table S2).

**Table 3:** Relative risks (RR) and vaccine effectiveness (VE) of Covid-19 vaccines on symptomatic, hospitalised, UCI admitted, and death by Covid-19 using the cohort of vaccinated indigenous people living in indigenous communities in Brazil

N = 370,092	Adjusted by age^a^		Adjusted by age and other covariates^b^	
	RR (95%CI)	VE (%) (95%CI)	RR (95%CI)	VE (%) (95%CI)
**Covid-19 incidence**				
*CoronaVac/AZ/Pfizer*				
1st dose (< 14 days)	1	.	1	.
1st dose ( > = 14 days)	0.45 (0.37–0.54)	55 (46–63)	0.49 (0.40–0.59)	51 (41–60)
2nd dose ( > = 14 days)	0.53 (0.45–0.63)	47 (37–55)	0.47 (0.40–0.56)	53 (44–60)
*CoronaVac*				
1st dose (< 14 days)	1	.	1	.
1st dose ( > = 14 days)	0.43 (0.36–0.52)	57 (48–64)	0.47 (0.39–0.57)	53 (43–61)
2nd dose ( > = 14 days)	0.48 (0.41–0.58)	52 (42–59)	0.46 (0.39–0.55)	54 (45–61)
**Covid-19 mortality**				
*CoronaVac/AZ/Pfizer*				
1st dose (< 14 days)	1	.	1	.
1st dose ( > = 14 days)	0.26 (0.06–1.08)	74 (-8-94)	0.26 (0.06–1.08)	74 (-9-94)
2nd dose ( > = 14 days)	0.47 (0.14–1.6)	53 (-60-86)	0.47 (0.14–1.56)	53 (-56-86)
*CoronaVac*				
1st dose (< 14 days)	1	.	1	.
1st dose ( > = 14 days)	0.26 (0.06–1.09)	74 (-9-94)	0.26 (0.06–1.09)	74 (-9-94)
2nd dose ( > = 14 days)	0.46 (0.14–1.53)	54 (-53-86)	0.46 (0.14–1.53)	54 (-53-86)
**Covid-19 hospitalisation**				
*CoronaVac/AZ/Pfizer*				
1st dose (< 14 days)	1	.	1	.
1st dose ( > = 14 days)	0.82 (0.34–1.96)	18 (-96-66)	0.82 (0.34–1.96)	18 (-96-66)
2nd dose ( > = 14 days)	0.59 (0.25–1.35)	42 (-35-75)	0.59 (0.25–1.35)	41 (-35-75)
*CoronaVac*				
1st dose (< 14 days)	1	.	1	.
1st dose ( > = 14 days)	0.99 (0.39–2.55)	1 (-155-61)	0.99 (0.39–2.55)	1 (-155-61)
2nd dose ( > = 14 days)	0.68 (0.27–1.69)	32 (-69-73)	0.68 (0.27–1.69)	32 (-69-73)
**Covid-19 progression to ICU** ^**3**^				
*CoronaVac/AZ/Pfizer*				
1st dose (< 14 days)	1	.	1	.
1st dose ( > = 14 days)	0.15 (0.02–0.98)	85 (2–98)	0.15 (0.02–0.93)	85 (2–98)
2nd dose ( > = 14 days)	0.13 (0.02–0.86)	87 (14–98)	0.14 (0.02–0.81)	87 (14–98)
*Coronavac*				
1st dose (< 14 days)	1	.	1	.
1st dose ( > = 14 days)	0.13 (0.02–0.86)	87 (14–98)	0.13 (0.03–0.79)	87 (14–98)
2nd dose ( > = 14 days)	0.12 (0.02–0.75)	88 (25–98)	0.12 (0.02–0.68)	88 (25–98)
**Covid-19 death among hospitalised patients** ^**c**^				
*CoronaVac/AZ/Pfizer*				
1st dose (< 14 days)	1	.	1	.
1st dose ( > = 14 days)	0.02 (0.01–0.08)	98 (92–99)	0.02 (0.01–0.08)	98 (92–99)
2nd dose ( > = 14 days)	0.04 (0.01–0.10)	96 (90–99)	0.04 (0.01–0.10)	96 (90–99)
*CoronaVac*				
1st dose (< 14 days)	1	.	1	.
1st dose ( > = 14 days)	0.02 (0.01–0.07)	98 (93–99)	0.02 (0.01–0.07)	98 (93–99)
2nd dose ( > = 14 days)	0.04 (0.01–0.10)	96 (90–99)	0.04 (0.01–0.09)	96 (91–99)

^a^Relative risks (RR) estimated using Poisson regression adjusted by age (continuous)

^b^Relative risks (RR) estimated using Poisson regression adjusted. RR for laboratory-confirmed cases was adjusted by age (continuous), sex, region, the month of the 1st dose vaccination, and municipal deprivation index (IBP); RR for mortality, hospitalisation, progression to ICU, and death were adjusted by age (continuous) and sex

^c^Among the 105 hospitalised cases

## Discussion

To our knowledge, this is the first study to evaluate vaccine effectiveness (VE) against Covid-19 in the Indigenous population in Brazil. Our results show that vaccination coverage is lower in the investigated indigenous people compared to the general Brazilian population. Two doses of any of the three vaccines (Coronavac, ChAdOx1, or BNT162b2) was at least 50% effective against symptomatic laboratory-confirmed Covid-19 cases and over 80% effective against progression to ICU and death within the hospital.

A previous study calculated vaccine coverage of indigenous groups in Brazil, pointing out that vaccine coverage in older adults was lower than in the general Brazilian population [23]. In our study, we also found lower coverage of partial or full vaccination against Covid-19 than in the general Brazilian population, with inadequate coverage (< 80%) for almost all strata of sex, region, socioeconomic index, and age. The low coverage in the North region (40.3%) is particularly worrying, given that it has the highest proportion of the indigenous population. Covid-19 vaccination coverage in Brazil has been marked by major structural socio-economic, environmental, and ethnic-racial inequities [24], with pronounced lower vaccination coverage than among the general Brazilian population in all but in the Northeast region of Brazil. Surprisingly, the indigenous vaccination coverage was similar between the North and South and Southeast regions, despite the two last areas being the most socio-economically developed and having the largest healthcare network in the country.

In Brazil, there were observed declining trends of the incidence and mortality caused by Covid-19 in indigenous people, which were supposed to have occurred due to the increased coverage of Covid-19 vaccines in that group during 2021. However, the study used aggregated data and did not provide a formal VE evaluation [23]. In the Colombian Amazon, which borders Brazil, CoronaVac showed over 94% effectiveness against symptomatic Covid-19 in a majority indigenous population of a municipality of 7856 inhabitants with very large (> 99%) vaccine coverage [25]. On the other hand, a similar population-based study using linked data have reported lower effectiveness of the Pneumococcal conjugate vaccine against all-cause pneumonia hospitalisations in indigenous peoples compared to their counterparts [26].

High VE against symptomatic and severe Covid-19 cases among the general Brazilian population has been previously shown using the same linked data sources [13]. In addition, by analysing VE for Covid 19 in Brazil, a similar study also observed that VE of two doses of CoronaVac was 54% for symptomatic infection and 74% for death in Brazil’s general population [17]. Our study found a similar VE for CoronaVac against symptomatic Covid-19 (54% in the indigenous people) but a lower VE against mortality (54%). The earlier study also analysed ChAdOx-1’s VE, finding a higher magnitude of protection against all outcomes compared to the VE conferred by CoronaVac [17]. These findings may be relevant to explain the lower magnitude of VE in the indigenous population when analysing the effect of the combined vaccination schedules since they had a higher proportion (87.0%) of vaccination with CoronaVac. In addition, our study indicated no reductions in the hospitalisation of indigenous vaccinated subjects, which differs from the pronounced VE levels for CoronaVac (72%) and ChAdOx-1 (87%) against hospitalisation in the general Brazilian population [17]. Finally, in our study, there was also a non-significant protective effect of either vaccine schemes (CoronaVac, ChAdOx-1, or BNT162b2) against deaths from Covid-19, regardless of the condition of hospitalisation, while Cerqueira-Silva et al. (2022) found 74% and 90% effectiveness, respectively, of CoronaVac and ChAdOx-1 against deaths in the general population of the country.

In interpreting our findings for indigenous populations, it is important to consider that 60.7% of them are located in Brazil’s Central-West and North regions. Indigenous territories in these regions are mostly situated in rural areas, with a scarcity of secondary and tertiary healthcare units in nearby towns. This results in major restrictions on access to specialised health care [15, 27]. During the peaks of the Covid-19 pandemic in 2020 and 2021, all regions in Brazil faced high demand for healthcare. The collapse of the Brazilian Unified Health System occurred particularly in cities in the most remote areas, such as in the North. These restraints in the provision of primary care by the HIS were also accompanied by the high circulation of fake news and vaccination hesitancy [28].

In addition, VE among the indigenous population in Brazil is likely to be influenced by lower vaccination coverage since high vaccination coverage in indigenous villages could have led to indirect protection of the community. Low Covid-19 vaccination coverage in many indigenous communities composed of just a few hundred individuals might also threaten their cultural continuity, as Covid-19 has higher mortality on older people who are largely responsible for cross-generational cultural transmission in these societies [14, 15]. Another possible explanation is the high rates of Covid-19 in indigenous communities prior to vaccination.

Our results shed light on the existing barriers of Indigenous Peoples in accessing healthcare services. Discrimination and social exclusion to which they have been historically submitted in the country reveal the need to expand the participation of the indigenous movement in the political struggle to pursue their constitutional rights and guarantee the strengthening of the HIS [10, 11, 13, 14]. Ensuring qualified and timely access to health, particularly vaccination and comprehensive healthcare, respecting social and cultural specificities, is key to mitigating the persistent inequalities in ethnic-racial morbidity and mortality due to Covid 19 in Brazil [10, 11, 13, 14].

It is important to mention some limitations of this study. Estimates of Covid-19 vaccination coverage depend on reliable population estimates. In the case of the indigenous population in Brazil, the most recent nationally representative demographic data was collected more than a decade ago, in the 2010 national census [29, 30]. A possible demographic source is the specific health information system of the IHS (known as SIASI - *Sistema de Informação da Atenção à Saúde Indígena*). Still, access to data in this system is not currently available to the general public, including researchers [31]. As an alternative, we estimated the 2020 indigenous population attended by IHS, applying the 2010 Census proportions of the indigenous population to the 2020 demographic estimates for the general population residing in the municipalities that overlap with the DSEI territories of the IHS. According to government data on Covid-19 [16], 657,758 indigenous individuals over 5 years of age were considered in the priority group for vaccination. These estimates differ by around 10% from our estimates of 599,540 individuals in the same age group. This difference could be related to the fact that we did not rely on demographic projections because variation in the indigenous population size in the recent Brazilian national censuses has been suggested to be largely affected by ethnic/racial classification issues and not solely by demographic dynamics [30]. This difference in the target population estimates might have led to an overestimated vaccine coverage, however, it did not impact the other findings of this study. We did not have sufficient power to evaluate waning and to stratify by calendar time, which would be necessary to investigate if VE among indigenous people is different from among non-indigenous groups. Also, as a small proportion of indigenous people had received the third dose by the time the data was extracted, we did not evaluate the VE of the third dose.

In addition, as data on Covid-19 vaccination in the indigenous population was only available from PNI, the only possible research design was a cohort study, taking vaccinated individuals (< 14 days of vaccination) as the control group. Using the cohort design, we certainly missed indigenous subjects living in urban areas not served by IHS, who have also been strongly affected by the pandemic but were not included as a priority group for vaccination. This implies restrictions on generalising our results beyond those indigenous living in municipalities overlapping with DSEI territories. Finally, IBP was estimated for the municipality, not for indigenous lands, and they probably differ even in the same municipality. Nevertheless, IPB still is an important indicator that could demonstrate differences in access to health services at a municipal level and their financial capacity to deal with the pandemic locally.

## Conclusions

Our results indicate low Covid-19 vaccine coverage among indigenous groups in Brazil but with similar VE to non-indigenous counterparts. The heterogeneity in vaccination coverage leaves clusters of indigenous populations particularly susceptible to Covid-19. Therefore, strengthening the IHS and supporting strategies to reduce health access barriers and expand vaccination acceptance and coverage against Covid-19, including booster doses, is key to preventing local outbreaks and reducing the unacceptable disproportionate impacts of Covid-19 on indigenous peoples.

## Electronic supplementary material

Below is the link to the electronic supplementary material.

Supplementary Material 1

## Data Availability

This dataset provided by the Brazilian Ministry of Health is not publicily available. However, the unidentified data underlying this article can be shared upon request to the authors and after ethical approval from The Brazilian National Commission on Research Ethics (CONEP). Please contact JMP (julia.pescarini1@lshtm.ac.uk) if you would like to have access to the data.

## List of abbreviations

IHS: Indigenous Health Care Subsystem
DSEIs: Special Indigenous Health Districts *(Distritos Sanitários Especiais Indígenas*)
VE: vaccine effectiveness
ICU: Intensive Care Unit
PNI: National Immunization Program
SI-PNI: Information System of Brazilian National Vaccination Programme
ILI: Influenza-like Illness
SARI: Severe Acute Respiratory Infection
IBP: Brazilian Deprivation Index (*Índice Brasileiro de Privação)*
IBGE: Brazilian Institute of Geography and Statistics

## References

[1] Walker RS, Sattenspiel L, Hill KR (2015). Mortality from contact-related epidemics among indigenous populations in Greater Amazonia. Sci Rep.

[2] Anderson I, Robson B, Connolly M, Al-Yaman F, Bjertness E, King A (2016). Indigenous and tribal peoples’ health (the Lancet–Lowitja Institute Global collaboration): a population study. The Lancet.

[3] Gracey M, King M (2009). Indigenous health part 1: determinants and disease patterns. The Lancet.

[4] Cardoso AM (2010). A persistência das infecções respiratórias agudas como problema de Saúde Pública. Cad Saude Publica.

[5] Cardoso AM, Resende PC, Paixao ES, Tavares FG, Farias YN, Barreto CTG (2019). Investigation of an outbreak of acute respiratory disease in an indigenous village in Brazil: contribution of Influenza A(H1N1)pdm09 and human respiratory syncytial viruses. PLoS ONE.

[6] Binks MJ, Beissbarth J, Oguoma VM, Pizzutto SJ, Leach AJ, Smith-Vaughan HC (2020). Acute lower respiratory infections in indigenous infants in Australia’s Northern Territory across three eras of pneumococcal conjugate vaccine use (2006–15): a population-based cohort study. Lancet Child Adolesc Health.

[7] Coimbra CEA (2014). Saúde e povos indígenas no Brasil: Reflexões a partir do I Inquérito Nacional de Saúde e Nutrição Indígena. Cad Saude Publica. Fundacao Oswaldo Cruz.

[8] Santos R, Welch J, Pontes A, Garnelo L, Cardoso A, Coimbra C Jr. Health of Indigenous Peoples in Brazil: inequities and the uneven trajectory of public policies. Oxford Research Encyclopedia of Global Public Health. Oxford University Press (forthcoming);; 2022.

[9] Montenegro RA, Stephens C (2006). Indigenous health in Latin America and the Caribbean. The Lancet.

[10] Hallal PC, Hartwig FP, Horta BL, Silveira MF, Struchiner CJ, Vidaletti LP (2020). SARS-CoV-2 antibody prevalence in Brazil: results from two successive nationwide serological household surveys. Lancet Glob Health.

[11] Lana RM, Codeço CT, Santos RV, Cunha B, Coelho FC, Cruz OG et al. 7 - Vulnerabilidade das populações indígenas à pandemia de Covid- 19 no Brasil e os desafios para o seu monitoramento. Covid-19 no Brasil: cenários epidemiológicos e vigilância em saúde. Série Informação para ação na Covid-19 | Fiocruz; 2021. p. 127–42.

[12] Soares GH, Jamieson L, Biazevic MGH, Michel-Crosato E (2022). Disparities in excess mortality between indigenous and non-indigenous brazilians in 2020: measuring the Effects of the COVID-19 pandemic. J Racial Ethn Health Disparities.

[13] Ranzani OT, Bastos LSL, Gelli JGM, Marchesi JF, Baião F, Hamacher S (2021). Characterisation of the first 250 000 hospital admissions for COVID-19 in Brazil: a retrospective analysis of nationwide data. Lancet Respir Med.

[14] Pontes A, Cardoso A, Bastos L, Santos R, Matta G, Rego S, Souto E, Segata J (2021). Pandemia de Covid-19 e os povos indígenas no Brasil: Cenários sociopolíticos e epidemiológicos. Os Impactos Sociais da Covid-19 no Brasil: Populações Vulnerabilizadas e Respostas à Pandemia.

[15] Boschiero MN, Palamim CVC, Marson FAL (2021). The hindrances to perform the COVID-19 vaccination in Brazil. Hum Vaccin Immunother.

[16] Brasil, PLANO NACIONAL DE OPERACIONALIZAÇÃO DA VACINAÇÃO. CONTRA A COVID-19. Brasília; 2022.

[17] Cerqueira-Silva T, Oliveira V, de Boaventura A, Pescarini VS, Júnior JM, Machado JB (2022). Influence of age on the effectiveness and duration of protection of Vaxzevria and CoronaVac vaccines: a population-based study. Lancet Reg Health - Americas.

[18] WHO. Interim statement on booster doses for COVID-19 vaccination. 2021.

[19] Brasil. Ministério da Saúde. Distrito Sanitário Especial Indígena. https://www.gov.br/saude/pt-br/composicao/sesai/estrutura/dsei.

[20] Cardoso A, Santos R, Garnelo L, Coimbra C, Chaves M, Giovanella L, Escorel S, Lobato L, Carvalho AI (2012). Políticas Públicas de Saúde para os Povos Indígenas. Políticas e Sistemas de Saúde no Brasil.

[21] Allik M, Ramos D, Agranonik M, Pinto Júnior EP, Ichihara MY, Barreto ML. Developing a Small-Area Deprivation Measure for Brazil: Technical Report [Internet]. 2020. Available from: https://cidacs.bahia.fiocruz.br/ibp/wp-content/uploads/2020/12/technical-report_compressed.pdf.

[22] Ranzani OT, Hitchings MDT, de Melo RL, de França GVA, Fernandes C, de FR, Lind ML (2022). Effectiveness of an inactivated Covid-19 vaccine with homologous and heterologous boosters against Omicron in Brazil. Nat Commun.

[23] Machado FCG, Ferron MM, Barddal MT da, Nascimento M, Rosalen LA, Avelino-Silva J. COVID-19 vaccination, incidence, and mortality rates among indigenous populations compared to the general population in Brazil: describing trends over time. Lancet Reg Health - Americas. 2022;13:100319. 10.1016/j.lana.2022.100319 PMC9294659 35874497

[24] Paim J, Travassos C, Almeida C, Bahia L, Macinko J (2011). The brazilian health system: history, advances, and challenges. The Lancet.

[25] Serrano-Coll H, Miller H, Guzmán C, Rivero R, Gastelbondo B, Miranda J (2022). Effectiveness of the CoronaVac® vaccine in a region of the colombian Amazon, was herd immunity achieved?. Trop Dis Travel Med Vaccines.

[26] Fathima P, Blyth CC, Lehmann D, Lim FJ, Abdalla T, de Klerk N (2018). The impact of pneumococcal vaccination on bacterial and viral pneumonia in western australian children: record linkage cohort study of 469589 births, 1996–2012. Clin Infect Dis.

[27] Damasco FS, Antunes M, Azevedo M, DESLOCAMENTOS DA POPULAÇÃO INDÍGENA, PARA ACESSO AOS SERVIÇOS DE SAÚDE. : ELEMENTOS PARA AÇÕES EMERGENCIAIS DE ENFRENTAMENTO À COVID-19. GEOgraphia. 2020;22.

[28] Galhardi CP, Freire NP, Fagundes MCM, Minayo MC, de Cunha S (2022). Fake news e hesitação vacinal no contexto da pandemia da COVID-19 no Brasil. Cien Saude Colet.

[29] IBGE. Censo Demográfico 2010: Características Gerais dos Indígenas: Resultados do Universo. Rio de Janeiro; 2010.

[30] Santos RV, Guimarães BN, Simoni AT, da Silva LO, de Oliveira Antunes M, de Souza Damasco F (2019). The identification of the indigenous population in Brazil’s official statistics, with an emphasis on demographic censuses. Stat J IAOS.

[31] Reis AC, Casanova AO, da Cruz MM, Cunha MLS, Gomes M, de F, Suárez-Mutis MC et al. Estudo de avaliabilidade do Sistema de Informação da Atenção à Saúde Indígena: potencialidades e desafios para apoiar a gestão em saúde no nível local. Cad Saude Publica. 2022;38. 10.1590/0102-311XPT021921 35584430

[32] Brasil. Ministério da Saúde. Cadastro Nacional de Estabelecimentos de Saude (CNES). Available at: http://cnes.datasus.gov.br/.

[33] Brasil. Ministério dos Povos Indígenas. Fundação Nacional dos Povos Indígenas (FUNAI). Geoprocessamento e Mapas. Available at: https://www.gov.br/funai/pt-br/atuacao/terras-indigenas/geoprocessamento-e-mapas.

