# Supplementary material for: Vaccine coverage and effectiveness against laboratory-confirmed symptomatic and severe Covid-19 in indigenous people in Brazil: a cohort study
Source: BMC Public Health. 2023 Jun 29;23:1267. doi: 10.1186/s12889-023-16196-4 (PMC10311776; doi:10.1186/s12889-023-16196-4)
Supplement: Supplementary file 1 — Supplementary Material 1 [file 12889_2023_16196_MOESM1_ESM.docx]

**SUPPLEMNTARY TABLES**

**Table S1.** Vaccine coverage among people aged 5 or more overall and among indigenous living in municipalities overriding DSEIs territories (target of priority for vaccination) according to sex, age, region, and deprivation index quintiles in Brazil up to 1st March 2022.

| **Characteristic** | **Indigenous people** | | | **Overall Brazilian people** | | |
| --- | --- | --- | --- | --- | --- | --- |
|  | **Estimated target population** | **Partial vaccination^a^**  **(N)** | **Full vaccination**^b^ **(N)** | **Estimated target population** | **Partial vaccination^a^ (N)** | **Full vaccination**^b^ **(N)** |
| **Total** | 599,540 | 389,753 | 291,702 | 197,025,392 | 172,613,433 | 147,440,287 |
| **Sex** |  |  |  |  |  |  |
| Woman | 294,647 | 193,289 | 146,070 | 101,034,534 | 90,268,715 | 78,338,015 |
| Men | 304,872 | 196,463 | 145,632 | 95,990,858 | 82,344,718 | 69,102,272 |
| **Age (years)** |  |  |  |  |  |  |
| 5-9 | 101,879 | 2,662 | 25 | 14,650,284 | 5,967,358 | 293,643 |
| 10-19 | 167,087 | 68,064 | 35,563 | 30,596,370 | 24,937,510 | 15,781,033 |
| 20-49 | 251,843 | 244,060 | 194,461 | 97,706,614 | 87,591,813 | 79,929,785 |
| 50-59 | 33,880 | 34,263 | 28,163 | 23,875,072 | 23,174,703 | 22,045,579 |
| >60 | 44,850 | 40,704 | 33,490 | 30,197,052 | 30,942,049 | 29,390,247 |
| **Region** |  |  |  |  |  |  |
| North | 295,908 | 168,247 | 119,164 | 15,275,581 | 12,921,116 | 11,258,040 |
| Northeast | 128,610 | 103,366 | 85,449 | 53,236,769 | 45,539,471 | 37,401,821 |
| Southeast | 32,144 | 15,386 | 13,195 | 17,048,634 | 13,015,438 | 10,366,288 |
| South | 49,174 | 33,866 | 21,143 | 83,235,426 | 74,761,969 | 64,841,366 |
| Central-West | 93,704 | 68,438 | 52,751 | 28,184,462 | 25,348,707 | 22,771,929 |
| **IBP quintiles** |  |  |  |  |  |  |
| 1 (less deprived) | 8,719 | 1,452 | 797 | 36,838,011 | 33,270,214 | 29,545,013 |
| 2 | 18,423 | 5,652 | 4,293 | 36,728,900 | 32,893,716 | 28,624,691 |
| 3 | 19,180 | 18,260 | 13,740 | 40,247,396 | 35,222,922 | 30,397,479 |
| 4 | 104,061 | 85,167 | 62,118 | 39,377,375 | 34,272,222 | 29,052,934 |
| 5 (more deprived) | 449,157 | 279,222 | 210,754 | 43,789,190 | 35,927,627 | 29,019,327 |

^a^Partial vaccination - one dose of ChAdOx-1, CoronaVac or Pfizer.

^b^Full vaccination - two doses of ChAdOx-1, CoronaVac or Pfizer; or one dose of Jannsen.

**Table S2.** Relative risks (RR) and vaccine effectiveness (VE) of Covid-19 vaccines on symptomatic, hospitalised, UCI admission and death Covid-19 cases using the cohort of vaccinated indigenous people living in indigenous communities in Brazil up to 31^st^ December 2021.

|  | **Adjusted by age^a^** | | **Adjusted by age and other covariates^b^** | |
| --- | --- | --- | --- | --- |
| **N=** **361,900** | **RR (95%CI)** | **VE (%) (95%CI)** | **RR (95%CI)** | **VE (%) (95%CI)** |
| **Covid-19 incidence** |  |  |  |  |
| *CoronaVac/AZ/Pfizer* |  |  |  |  |
| 1st dose (<14 days) | 1 | ·· | 1 | ·· |
| 1st dose (>=14 days) | 0·49 (0·40-0·59) | 51 (41-60) | 0·50 (0·42-0·61) | 50 (39-58) |
| 2nd dose (>=14 days) | 0·53 (0·45-0·63) | 47 (37-55) | 0·46 (0·39-0·55) | 54 (45-61) |
| *CoronaVac* |  |  |  |  |
| 1st dose (<14 days) | 1 | ·· | 1 | ·· |
| 1st dose (>=14 days) | 0·46 (0·38-0·56) | 54 (44-62) | 0·49 (0·41-0·60) | 51 (40-59) |
| 2nd dose (>=14 days) | 0·49 (0·41-0·59) | 51 (41-59) | 0·46 (0·39-0·55) | 54 (45-61) |
| **Covid-19 death** |  |  |  |  |
| *CoronaVac/AZ/Pfizer* |  |  |  |  |
| 1st dose (<14 days) | 1 | ·· | 1 | ·· |
| 1st dose (>=14 days) | 0·29 (0·07-1·22) | 71 (-22-93) | 0·29 (0·07-1·22) | 71 (-22-93) |
| 2nd dose (>=14 days) | 0·49 (0·15-1·62) | 51 (-62-85) | 0·49 (0·15-1·63) | 51 (-63-85) |
| *CoronaVac* |  |  |  |  |
| 1st dose (<14 days) | 1 | ·· | 1 | ·· |
| 1st dose (>=14 days) | 0·29 (0·07-1·22) | 71 (-22-93) | 0·29 (0·07-1·23) | 71 (-23-93) |
| 2nd dose (>=14 days) | 0·48 (0·15-1·60) | 52 (-60-85) | 0·48 (0·15-1·60) | 52 (-60-85) |
| **Covid-19 hospitalisation** |  |  |  |  |
| *CoronaVac/AZ/Pfizer* |  |  |  |  |
| 1st dose (<14 days) | 1 | ·· | 1 | ·· |
| 1st dose (>=14 days) | 0·93 (0·39-2·22) | 7 (-122-61) | 0·93 (0·39-2·22) | 7 (-122-61) |
| 2nd dose (>=14 days) | 0·61 (0·26-1·41) | 39 (-41-74) | 0·61 (0·26-1·41) | 39 (-41-74) |
| *CoronaVac* |  |  |  |  |
| 1st dose (<14 days) | 1 | ·· | 1 | ·· |
| 1st dose (>=14 days) | 1·11 (0·43-2·86) | -11 (-186-57) | 1·12 (0·43-2·86) | -12 (-186-57) |
| 2nd dose (>=14 days) | 0·71 (0·29-1·78) | 29 (-78-71) | 0·71 (0·29-1·78) | 29 (-78-71) |
| **Covid-19 progression to ICU^3^** |  |  |  |  |
| *CoronaVac/AZ/Pfizer* |  |  |  |  |
| 1st dose (<14 days) | 1 | ·· | 1 | ·· |
| 1st dose (>=14 days) | 0·15 (0·02-0·99) | 85 (1-98) | 0·15 (0·02-0·96) | 85 (4-98) |
| 2nd dose (>=14 days) | 0·15 (0·02-0·95) | 85 (5-98) | 0·15 (0·02-0·91) | 85 (9-98) |
| *CoronaVac* |  |  |  |  |
| 1st dose (<14 days) | 1 | ·· | 1 | ·· |
| 1st dose (>=14 days) | 0·13 (0·02-0·86) | 87 (14-98) | 0·13 (0·02-0·82) | 87 (18-98) |
| 2nd dose (>=14 days) | 0·13 (0·02-0·83) | 87 (17-98) | 0·13 (0·02-0·78) | 87 (22-98) |
| **Covid-19 progression to death in hospitalised patients^c^** |  |  |  |  |
| *CoronaVac/AZ/Pfizer* |  |  |  |  |
| 1st dose (<14 days) | 1 | ·· | 1 | ·· |
| 1st dose (>=14 days) | 0·02 (0·01-0·08) | 98 (92-99) | 0·02 (0·01-0·08) | 98 (92-99) |
| 2nd dose (>=14 days) | 0·04 (0·01-0·1) | 96 (90-99) | 0·04 (0·01-0·1) | 96 (90-99) |
| *CoronaVac* |  |  |  |  |
| 1st dose (<14 days) | 1 | ·· | 1 | ·· |
| 1st dose (>=14 days) | 0·02 (0·01-0·07) | 98 (93-99) | 0·02 (0·01-0·07) | 98 (93-99) |
| 2nd dose (>=14 days) | 0·04 (0·01-0·1) | 96 (90-99) | 0·04 (0·01-0·1) | 96 (90-99) |
| **Covid-19 death** |  |  |  |  |
| *CoronaVac/AZ/Pfizer* |  |  |  |  |
| 1st dose (<14 days) | 1 | ·· | 1 | ·· |
| 1st dose (>=14 days) | 0·29 (0·07-1·22) | 71 (-22-93) | 0·29 (0·07-1·22) | 71 (-22-93) |
| 2nd dose (>=14 days) | 0·49 (0·15-1·62) | 51 (-62-85) | 0·49 (0·15-1·63) | 51 (-63-85) |
| *CoronaVac* |  |  |  |  |
| 1st dose (<14 days) | 1 | ·· | 1 | ·· |
| 1st dose (>=14 days) | 0·29 (0·07-1·22) | 71 (-22-93) | 0·29 (0·07-1·23) | 71 (-23-93) |
| 2nd dose (>=14 days) | 0·48 (0·15-1·60) | 52 (-60-85) | 0·48 (0·15-1·60) | 52 (-60-85) |

^a^Relative risks (RR) estimated using Poisson regression adjusted by age (continuous).

^b^Relative risks (RR) estimated using Poisson regression adjusted. RR for incidence adjusted by age (continuous), sex, region, the month of the 1^st^ dose vaccination and municipal deprivation index (IBP); RR for hospitalisation, ICU and death adjusted by age (continuous) and sex.

^c^Among the 100 hospitalised cases.
